# Contributions of the International Narcotics Research Conference to Opioid Research Over the Past 50 years

**DOI:** 10.3389/adar.2022.10115

**Published:** 2022-02-18

**Authors:** Brian M. Cox, Lawrence Toll

**Affiliations:** ^1^ Department of Pharmacology and Molecular Therapeutics, Uniformed Services University of the Health Sciences, Bethesda, MD, United States; ^2^ Department of Biomedical Sciences, Charles E. Schmidt College of Medicine, Florida Atlantic University, Boca Raton, FL, United States

**Keywords:** opioid, INRC, opiate receptor, endorphin, opioid discoveries

## Abstract

The International Narcotics Research Conference (INRC), founded in 1969, has been a successful forum for research into the actions of opiates, with an annual conference since 1971. Every year, scientists from around the world have congregated to present the latest data on novel opiates, opiate receptors and endogenous ligands, mechanisms of analgesic activity and unwanted side effects, etc. All the important discoveries in the opiate field were discussed, often first, at the annual INRC meeting. With an apology to important events and participants not discussed, this review presents a short history of INRC with a discussion of groundbreaking discoveries in the opiate field and the researchers who presented from the first meeting up to the present.

## Introduction

**FIGURE 1:**
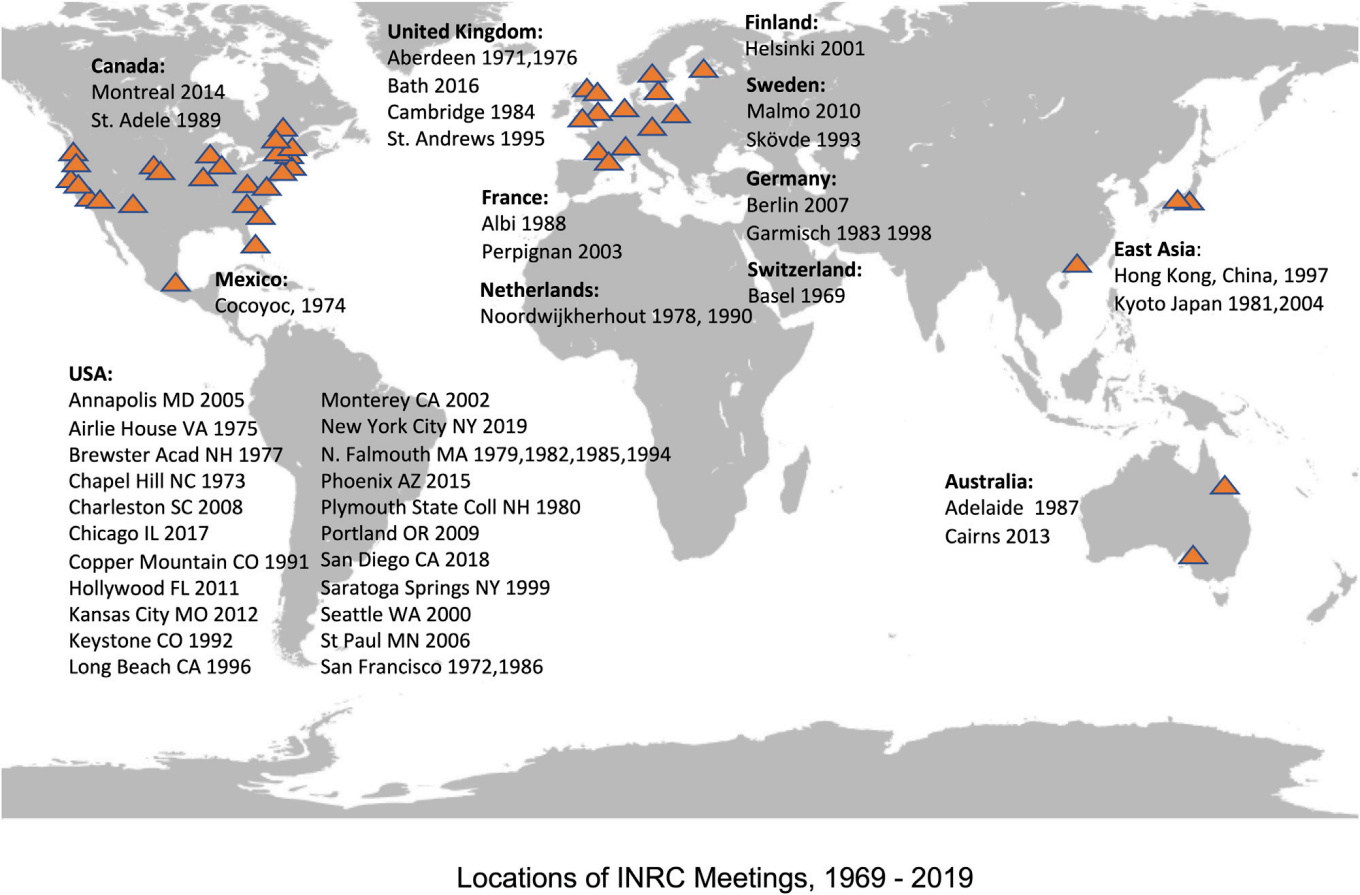
Location of INRC Meetings, 1969–2019 Red triangles indicate the locations of individual INRC meetings. INRC has met at some of these locations on more than one occasion.

INRC (the International Narcotics Research Conference; originally called a “Club”), founded in 1969, has now been a successful annual conference for those active in research into the actions of opiate drugs and the functional roles of endogenous opioids for over 50 years, holding annual meetings at sites across the world from 1971 to 2019. The planned 2020 meeting was canceled because of the restrictions on meetings and travel occasioned by the Covid-19 pandemic; on-line virtual meetings were held in July 2020 and 2021. The INRC will return to in-person meetings in 2022, with an exciting meeting to be held in July in Valencia, Spain. Figure 1 depicts the locations of INRC meetings across the world during its first 50 years. The majority of these meetings were held in North America or Europe because a majority of active opioid research groups are in these locations, but over the 50-years period five meetings have been held in the Far East and in Australia and one in Central America. A full list of the meeting sites for all INRC meetings, Presidents and Program Chairs is presented on the INRC website, located at www.inrconference.org.

The goal of the Conference is to create an annual forum for the presentation of cutting-edge research on opioids, encompassing the pharmacology of opioid drugs, the physiological roles of endogenous opioids and the receptor systems they regulate, the mechanisms underlying addiction, tolerance and dependence of opioid drugs, and on research guiding the optimal therapeutic applications of opioids and agents designed to modulate the functions of endogenous opioid systems for therapeutic benefit. Another important goal has been in support and encouragement of young scientists entering the field including offering a venue for presentation of their work to the international community. To do this, INRC has throughout its history offered travel awards to young scientists to enable them to attend and to present at its meeting. This has been possible largely because, for 40 years or more, the US National Institute on Drug Abuse (NIDA) has provided funding to cover travel awards to young scientists from the United States and local organizers of meetings outside the United States have been very successful in raising funds to support meeting attendance from their local communities.

Early in its history, INRC adopted a set of By-Laws outlining the principles governing the organization and the planning of meetings. These established an Executive Committee with two representatives from each of the three regions, North Americas, Europe, and the Rest of the World, elected, during a Business Meeting held at each Conference, to 4-years terms of service to the Conference. For almost the entire 50-years period, membership was attained simply by attendance at an INRC meeting (The Conference only became a formal membership organization with a membership fee in 2020). Proposals for future meetings are presented to members at each Business Meeting, and the choice is made by ballot during the meeting. Meeting locations have been selected in part on the professional locations of attendees. Generally, over the first 50 years, two consecutive meetings have been held at appropriate locations in the United States, with the next meeting in Europe or the Rest of the World, but this arrangement has been flexible enough to permit the Conference to meet on occasion in the same location as other groups such as the World Congress of Pharmacology. For much of its existence the leadership style was very informal. A Secretary was appointed initially from the group of founding members and later from the more senior active INRC participants to oversee meeting continuity and to work with a local organizing committee to plan the content of each meeting. The title of Secretary was later changed to President to reflect more clearly the functions associated with this position.

In the early years, meeting programs contained a few talks by senior leaders in the field selected by the President and the meeting organizing committee and short talks and posters selected from submitted abstracts. Programs were organized around general themes such as receptors, endogenous ligands, signal transduction, *in vivo* studies of drug action, and the relationship between tolerance, dependence and addiction. As the need to develop more complex stories arose and as meeting attendance grew, the number of invited speakers expanded, although organizing committees continued to invite presentations from younger scientists making novel contributions to the field. In the early 2000s, under the Presidency of Charles Chavkin, meeting organizers also decided to include a formal lecture by one of the founding members of INRC, naming this lecture the Founders Lecture, following a suggestion by Alan North. In later years the honor of presenting a Founders Lecture has been awarded to many senior contributors to the field of opioid research, all of whom have been important contributors to INRC. It has also become a tradition to present a plenary lecture by a local scientist who has made major contributions in a related field of biomedical research but outside the world of opioid pharmacology, with the goal of encouraging the membership to think constructively about widening the scope of the field.

### Early History

A brief account of the founding of the INRC, written with characteristic informality by Sydney Archer, one of the founders and a former Secretary of INRC, appears on the INRC Website (under “About Us”). The occasion was a meeting of the 1969 World Congress of Pharmacology during which Hans Kosterlitz and Harry Collier chaired a short satellite meeting on the pharmacology of opioid drugs. Kosterlitz and Collier had chosen a small but select international group of speakers for this meeting, including Sydney Archer and Avram Goldstein from the United States, Albert Herz from Germany, as well as Kosterlitz and Collier from the UK, balancing speakers from the academic and private sectors (Archer and Collier both had long careers in the private sector). All were well established scientists with a record of contributions to research on opioids. Kosterlitz, who had left Germany following the Nazi rise to power, arrived in Aberdeen in 1934, initially obtaining a junior research position in the medical school, but he rose quickly through the academic ranks, eventually becoming Professor and Head of the Pharmacology Department. By the late 1960s he had an international reputation for his studies of drug actions in the autonomic and enteric nervous systems and had demonstrated that the actions of morphine-like drugs on transmitter release in the guinea pig isolated ileum preparation could be used to predict their potential addictive liability. On the basis of these studies, he had been awarded a contract with the Committee on Problems of Drug Dependence (CPDD) to evaluate novel synthetic and semi-synthetic opioid drugs for their addictive liability using his bioassay systems. Harry Collier had a varied career, mainly in the pharmaceutical industry where he had a long-term interest in the identification of novel agents for the relief of pain. By the late 1960s he had left his Research Director position at Parke Davis, UK, and moved to become Director of Research at Miles Laboratories, UK. Sydney Archer was an organic chemist who spent almost his entire career at Sterling-Winthrop in Rensselaer NY, United States. He attained the position of Vice President at Sterling-Winthrop while concurrently holding the position of Professor of Medicinal Chemistry (and ultimately Dean) at Rensselaer Polytechnical Institute. By the late 1960s he had already published extensively on the medicinal chemistry of morphine and many series of analogues and was particularly associated with the development and marketing of pentazocine and other benzomorphans. Avram Goldstein, who was the founding Chair of the Department of Pharmacology at Stanford University in California and who had recently (in 1968) founded the journal *Molecular Pharmacology*, began working on the pharmacology of opioids in the 1960s, after earlier work on mechanisms of inhibition of cholinesterases and in the field of bacterial genetics. By 1969 he had already published several papers extending quantitative techniques to the evaluation of varied actions of opioid drugs. Albert Herz’ research career was largely spent at the Max Planck Institute for Psychiatry in Munich, Germany. By 1969, he was Head of the Department of Neuropharmacology, where he directed a multidisciplinary group, including pioneering studies using electrophysiological recordings together with functional assays to evaluate specific target sites for morphine within the brain and spinal cord. According to Syd Archer’s account of the World Congress meeting, the proceedings were “lively and interesting,” but there is no extant record of the presentations at the Congress.

After the symposium the speakers moved to the nearby Hotel Euler for dinner. It was during this dinner that it was agreed to convene follow-up meetings in subsequent years. The choice of a name for the new conference generated discussion. “International” was a given. The American speakers wanted to include the term “narcotics” for opioid drugs since this drug class was then commonly called by that term in the United States and by the World Health Organization; this application of the term derives from its legal use in the United States, including in several acts of the U.S. Congress seeking to limit access to addictive opioids, dating as far back as the early 1900s. The Founders decided on the name “club” rather than “conference,” in keeping with their sense that the most useful exchanges of views on research among scientists occurred in highly informal settings, and this informality has been a feature and goal of the INRC ever since. Hence the name International Narcotics Research Club (INRC). It may be no coincidence that a widely attended regular conference on the physiology and pharmacology of catecholamines, commonly called the “Catecholamine Club,” was founded in 1968, just before the founding of INRC (The name for INRC was later changed from Club to Conference, in part because NIDA became very reluctant to provide financial support to a “Club” amid the potential concern that membership in a Club might be viewed as selective and discriminatory).

Organization of the meetings of the Club/Conference was initially based on invitations from senior scientists who volunteered to organize and manage individual meetings. Hans Kosterlitz volunteered to organize the second meeting of the group in Aberdeen, Scotland in 1971, and he was successful in getting financial support for this meeting from the British Pharmacological Society. However, a condition of the support on this occasion was that an account of the proceedings should be published. The resulting publication (1) provides a record of the state of the field at this early point in the history of INRC. There were 29 oral presentations over 3 days of meeting, mostly from United States and United Kingdom contributors. Only two of the 29 presentations (about 7%) were by women, and one of these, by Marthe Vogt, a major contributor to the world of serotonin and catecholamine physiology and pharmacology but with limited experience in the field of opioids, was essentially an introduction to subsequent talks on the interactions of opioids with the classical neurotransmitter systems (about 11 talks on this topic, and approximately 38% of the presentations at the meeting). Six of the presentations at the meeting (about 21%) were from research groups in the pharmaceutical industry, focused on synthetic opioids in the benzomorphan series and on the newly discovered oripavine derivatives (including etorphine). Two presentations reported on opioid drug effects in non-human primates (both on rhesus monkeys), and four presentations discussed opioid action in human subjects, (about 14% of presentations), including reports on pain relief in cancer patients, but none of the clinical studies reported randomized blinded clinical trials. Although the use of methadone as a treatment for heroin addiction had been introduced by Vincent Dole, Marie Nyswander and Mary Jeanne Kreek in 1965, by 1971 this was still not a widely used approach for the treatment of heroin addicts and there was no discussion of the use of chronic legal opioid agonist therapies to reduce relapse in heroin addicts during the 1971 meeting. It should be noted that terminology relating to the pharmacology of drugs derived from the opium poppy and the receptors through which they regulate biological systems and their endogenous ligands has changed over the five decades of INRC. Here we use the term “opioid” to refer to the natural and synthetic ligands of the receptors, also called collectively “opioid.” The term “opiate” will be used to describe agents directly derived from the natural ligands for these receptors (e.g., morphine, codeine) that are produced by the opium poppy.

Six presentations in the Aberdeen meeting were primarily directed to characterizing the “morphine” receptor. Since morphine was a plant alkaloid not found in mammalian tissues, the concept was controversial but had become generally accepted in the two preceding decades, largely on the basis of structure-activity studies evaluating analgesic responses to several series of morphine analogs that demonstrated the strict structural requirements for activation of the proposed receptor. William “Bill” Martin from the Lexington Addiction Research Center went further, presenting his concept of “receptor dualism,” a theoretical model postulating the existence of “distinguishable independent categories of receptors which induce analgesia” to explain the observation that some morphine analogs antagonized the analgesic actions of morphine at low doses while producing analgesic effects at higher doses. Kosterlitz’ presentation included a detailed account of the pharmacology of opioids in the isolated guinea pig ileum preparation. Other reports included structure-activity analyses in different series of opioid structures in specific assay systems. By 1970, radiolabeled drugs were being used increasingly by pharmacologists in their attempts to identify the elusive receptors for neurotransmitters and hormones. Some initial success had been obtained in the search for steroid hormone receptors and for nicotinic cholinergic receptors, but at this date direct measurement of any transmitter or hormone receptor by quantifying their binding of ligands was still very problematic. The challenge in using radio-labelled ligands to identify opioid receptors was that the receptors were expressed at very low levels relative to the levels of most other cellular constituents, making it very difficult to discriminate the very small amount of ligand bound to the receptor from the much larger quantity of irrelevant binding at lower affinity but high capacity to many other tissue constituents. It was at the Aberdeen meeting of INRC that Avram Goldstein proposed that the very marked stereoselectivity that had been previously observed in studies of the analgesic actions of several series of morphine analogs could be used to discriminate readily saturable binding to the receptors from the much larger pool of non-specific binding by measuring the binding of very low concentrations of radiolabeled levorphanol in the presence of a large excess of its *dextro*-rotatory isomer. The *d*-isomer, dextrorphan, did not produce analgesia *in vivo*, and was therefore presumed not to bind to the receptor, while still binding non-specifically to other tissue constituents in a manner analogous and quantitatively similar to its *levo*-rotatory isomer levorphanol. In Goldstein’s experiments reported at the meeting, specific receptor binding was still an exceedingly low percentage of the non-specific binding, but the concept that stereospecificity could be used to permit the identification of receptors for opioids was established (2). Despite the strong interest in ways to identify the receptors mediating the action of opioid drugs, the issue of whether these presumed receptors were physiologically regulated by an endogenous agent (i.e., not only by a plant product or synthetic chemical) was not discussed in any of the formal sessions of the meeting, but it was a recurrent subject of informal conversation among attendees at the meeting.

The success of the Aberdeen meeting, which was very well attended, encouraged the Founders to develop somewhat more formal mechanisms for planning for future meetings. Avram Goldstein agreed to serve as Secretary for the Club, with the general agreement that the Secretary would take on the overall management of the club over a period of 4 years, with yearly meetings to be planned together with local hosts at sites across the globe where there was a group of researchers with active opioid research programs who could offer a meeting venue that would be attractive for both local and international attendees. The Secretary would provide general oversight while the local organizers would make local arrangements and encourage local participation in the meeting, ideally with financial support generated locally. The first INRC meeting in 1969 had been held in conjunction with a World Congress of Pharmacology (WCP), and it was agreed that the 1972 meeting of INRC would be held as a satellite meeting of the WCP meeting in San Francisco United States, in that year. This being home turf for Avram Goldstein, he organized the 1972 meeting, inviting an international list of speakers while also including talks from several groups from the West Coast of the United States who were working on opioid drugs, establishing the idea that INRC meetings would promote talks by local research groups as well as from internationally established groups. Plans were made for the 1973 meeting in Chapel Hill, North Carolina (1973), and in 1974 INRC met in Cocoyoc, Mexico, at the invitation of J.E. Villareal, then working at the University of Michigan but also working with Miles Laboratories in Mexico, in collaboration with a local organizing committee. This meeting was planned as a satellite meeting of the annual meeting of CPDD in Mexico City the week before the INRC meeting, an arrangement that would be followed on a number of occasions in future years.

### The Search for the Opioid Receptor and its Endogenous Ligand

In keeping with the goal of encouraging presenters to report on-going cutting-edge research, the proceedings of the 1972, 1973 and 1974 meetings were not recorded by publication as journal papers or a monograph, since it was thought these short reports would block a full publication and therefore people would be hesitant to report ongoing research in this rapidly advancing field. Accordingly, copies of the programs of these meetings are not available (to the knowledge of the authors). However, studies on identification of what was then called “the opiate receptor” (since with the exception of Bill Martin’s group, most at that time assumed there would only be one such receptor) were rapidly advancing by this time. In early 1973 Candace Pert and Solomon Snyder of The Johns Hopkins University (3) published their ground-breaking study in *Science*, demonstrating the high-affinity binding of tritium-labelled naloxone in rat brain membranes, and this was fairly rapidly followed by reports from Eric Simon and others from New York University (4) of tritium labeled etorphine binding, and of tritium labeled dihydromorphine to brain membranes from Lars Terenius at the University of Uppsala, Sweden (5). These results and their ongoing studies after the initial publications were presented in person at the 1973 and 1974 meetings, along with reports from collaborators using varied techniques to evaluate the locations of expression and the properties of the receptors. Both Eric Simon and Lars Terenius became regular attendees at INRC meetings, and Eric Simon served for 4 years as Secretary/President of the Conference (1980–1983). The initial discoveries of the receptor were rapidly followed by more advances. Michael Kuhar and others in the Snyder group used autoradiography to map brain regions showing high affinity opioid receptor binding, providing the first description of the distribution of the receptors across the brain (6), and Gavril Pasternak and others from the same laboratory evaluated the chemical properties and ionic regulation of the binding sites, demonstrating the ability of sodium ions at physiologically relevant intracellular concentrations to increase the affinity of the binding site for an antagonist (naloxone) while decreasing the affinity of agonists such as morphine, thereby identifying a simple *in vitro* binding method to discriminate agonists and antagonists (7). The sodium sensitivity of opioid receptors was much later determined to be a common feature of many G protein coupled receptors that signaled through the inhibitory proteins G_i_ or G_o_. In each of these studies, very high affinity and stereochemical specificity was used to identify receptor binding, along with displacement studies comparing the relative potencies of series of opioid and non-opioid drugs and metabolites. In addition to stereospecificity, the key to observing receptor binding was the use of very high-specific activity radioligands, which in the case of opioids were not available prior to the early 1970s, and the use of rapid “washing” of the membrane-radioligand complexes with cold buffer to remove most of the non-specific binding and thereby permitting the reliable quantification of the very small fraction of high affinity binding.

The demonstration of opioid drug binding to high affinity tissue sites located in brain regions associated with opioid drug actions confirmed the earlier predictions that these drugs acted through specific receptors, leading to predictions that these receptors must normally be activated by an endogenous factor. Evidence in support of this concept included the demonstration in 1971 by John Liebeskind and his colleagues at UCLA (including David Mayer and Huda Akil) that stimulation of discrete regions of rat brain induced an analgesic effect that persisted longer than the duration of electrical stimulation, suggesting that the electrical stimulus induced the release of an endogenous agent acting like opioid drugs (8, 9). Later, Akil and others showed that there was partial analgesic cross-tolerance between morphine and brain stimulation, and the analgesia induced by electrical stimulation was antagonized by naloxone (10). There were no formal reports of studies seeking endogenous ligands for the receptors binding opioid drugs during the 1974 INRC meeting in Cocoyoc, Mexico, but at the end of the meeting Hans Kosterlitz hinted during the closing discussion that his laboratory was close to reporting on an endogenous agent that activated opioid receptors.

The 1975 INRC meeting was held at Airlie House, Virginia, just west of Washington DC, again immediately following a meeting of CPDD in Washington. Interest in the scientific community in opioid receptor research was increasing rapidly by this time, and the journal *Life Sciences* expressed interest in publishing reports of the proceedings rapidly after the meeting; speakers were asked to bring “camera-ready” copies of their papers to the meeting to facilitate quick publication in special issues of the journal [*Life Sci,* vols 16 (12) and 17 (1)]. These papers were later published by Pergamon Press together as a separate bound volume with Avram Goldstein as editor (11). By 1975, John Hughes and Hans Kosterlitz were ready to present their data on an endogenous opioid ligand for the opioid receptor, and the first paper at the 1975 meeting was their report showing that an endogenous peptide-like material extracted from pig brain, which they named enkephalin, was a potent activator of receptors in the mouse isolated vas deferens preparation that could be antagonized by naloxone with a similar pK_a_ to that observed for naloxone antagonism of normorphine. However, they had not yet determined the amino acid sequence of enkephalin. By this time other groups, including the Terenius group and the Snyder group had partially isolated from brain extracts materials (structures still unidentified) that could displace radiolabeled opioid ligands from high affinity opioid binding sites in brain membranes, and their reports immediately followed the Hughes and Kosterlitz paper (12–14). In the same session the Goldstein group reported on two pituitary fractions containing peptide-like materials (readily destroyed by proteolytic enzymes) from extracts of bovine and porcine pituitaries that were potent, naloxone-sensitive inhibitors of the isolated guinea pig ileum and mouse vas deferens preparations, and it was immediately apparent that these materials showed onset and recovery rates in the bioassay preparations that differed from the properties of the enkephalin fraction, as described by Hughes and Kosterlitz. Receptors also occupied a significant fraction of the program; the second and third sessions at the 1975 meeting included reports from several groups, including those of Snyder, Simon, Goldstein, Herz, Theodore Brody (Michigan State University), Jose Musacchio (NYU) and Horace Loh and E.L. Way (UCSF), covering their latest studies on the properties of the opioid ligand binding sites in brain membranes.

Signal transduction mechanisms were not neglected. A significant breakthrough in understanding downstream signaling from the opioid receptor had occurred in the previous year, when Harry Collier had reported (15) that morphine and other opioid drugs inhibited the activity of the enzyme adenylyl cyclase (AC) in brain membranes after it had been stimulated by prostaglandins. Collier now expanded on this report and suggested that inhibition of AC could account for the analgesic and other effects of these drugs, and furthermore that dependence on opioids might arise as a result of a “biochemical hypertrophy” of this system. Werner Klee and Marshall Nirenberg had shown in 1974 that neuroblastoma X glioma hybrid cells (NG108-15 cells) also expressed a receptor binding opioid drugs and that activation of this receptor inhibited AC activity and reduced cAMP levels in these cells, while chronic exposure to morphine over 2–4 days resulted increased AC activity (16, 17). Similar results were also reported at the meeting by Bernd Hamprecht and others (from the Max Planck Institüt for Biochemie, Martinsreid, Germany). Other talks in the session further examined the effects of opioid drugs on brain nucleotides and the possible roles of cyclic nucleotides and phosphodiesterases in opioid tolerance and dependence. The meeting also included accounts of the actions of opioids evaluated electrophysiologically in single neurons, in the enteric and autonomics nervous systems (from Ray Dingledine, Stanford; and Alan North & Graeme Henderson, Aberdeen), in spinal cord (Hiroshi Takagi, Kyoto; Jean-Marie Besson, Paris) and in brain (M Satoh & W Zieglgänsberger, Munich; Robert Frederickson, Lilly, Indianapolis). Another session at the meeting was devoted to ongoing studies of opioid drug interactions with classical neurotransmitter systems and their roles in the analgesic and tolerance/dependence-inducing actions of opioids.

Thus, by mid-1975 the general themes that would occupy the main focus of attention at future INRC meetings for the next forty-plus year were already in place, albeit at a very preliminary level, with new developments in each area and an increasing understanding of the complexity of both the endogenous opioid systems and the receptor systems through which they acted evolving rapidly over this period, as new technological approaches became available. Syd Archer took over the role of INRC Secretary in 1976 from Avram Goldstein, establishing the usual 4-years term for the Secretary/President. Over the following years, INRC meetings were held in Aberdeen, Scotland again (1976), Brewster Academy, New Hampshire, United States (1977—as a Gordon Conference), Noordwijkerhout, Netherlands (1978), Seacrest Hotel, N. Falmouth, on Cape Cod, Massachusetts (1979), and Plymouth State College, New Hampshire (1980, again as a Gordon Conference). Two of these locations proved particularly popular; INRC returned to Noordwijkerhout again in 1990, and to Seacrest in 1982, 1985 and 1994.

### Identification of Endogenous Opioids

The first important development following the 1975 Airlie House meeting was the publication in Nature in late 1975 of the chemical structures of [Met^5^]enkephalin (ME) and [Leu^5^]enkephalin (LE) (12). The unexpected presence of two closely related peptides in their active fractions had complicated their chemical identification; this was eventually achieved by the Kosterlitz group using mass spectrometry, the first time this technique had been used to sequence a neuropeptide. When the sequence of ME was reported it was immediately recognized by groups working on previously characterized peptides in the pituitary that the ME sequence formed part of the C-terminus of the pituitary peptide, β-lipotropin, previously sequenced by CH Li (at UCSF, San Francisco) (18), and by Derek Smyth (MRC, Mill Hill, London) (19). The opioid-like activity of the C-terminus of β-lipotropin was rapidly confirmed by several groups. β-Lipotropin itself was also recognized as being derived from a yet large precursor peptide that also yielded melanocyte stimulating hormone (α-MSH) and adrenocorticotrophin (ACTH). Accordingly, this precursor was given the name pro-opiomelanocortin (POMC). Careful analysis of the opioid activities isolated from pituitary by the Goldstein group indicated that one of the previously reported active fractions that he had isolated contained β-endorphin, but the nature of the other highly basic active fraction remained uncertain for several years, only being identified later as the novel opioid, dynorphin. With the discovery of more than one endogenous peptide capable of activating opioid receptors, a general term for this class of peptide was needed. Eric Simon proposed in 1976 that the term “endorphins” should be used as the collective noun covering all endogenous peptides that activated opioid receptors, and this term is now widely used, both by neuroscientists and by the general public.

The concept that receptors through which morphine and related drugs produced analgesia were normally regulated by hitherto unknown, but now newly identified endogenous neuropeptides, was first discussed at INRC, and electrified the field. While the enkephalins and other endogenous opioids were not the first neuropeptides to be discovered (substance P, oxytocin and vasopressin, and peptides regulating hypothalamic hormones were identified earlier), they were the first neuropeptides to be discovered through a search for the ligands/activators of receptors previously defined only by their sensitivity to exogenous drugs. This greatly increased interest in searching for other functional neuropeptides, and drove numerous technical advances in methods to find, localize, quantify, and evaluate the actions of other previously unstudied neuropeptides from brain and other tissues. The prior existence of a wide range of chemically different opioid drugs was a major factor in permitting the ligand binding properties of opioid receptors to be fully characterized, and receptor characterization was further facilitated by analysis of their interaction with the wide range of opioid peptides now being discovered.

The gene coding for POMC was cloned and sequenced by the Shosaku Numa group (in Kyoto, Japan) in 1979 (20), the first of a series of remarkable achievements in which the Numa group also cloned and characterized the genes for the enkephalin and dynorphin precursor peptides (see below). The LE sequence is not part of POMC and the origins of LE remained uncertain for a few more years. Extended ME-containing peptides (including ME-Arg-Phe and ME-Arg-Arg, and longer extended peptides) also not contained in the β-lipotropin structure were later isolated from adrenal gland and other tissues, suggesting that POMC was unlikely to be the only ME precursor or even its primary precursor. In the years following the initial report of the enkephalins, several groups reported at INRC meetings between 1976 and 1981 that various extended enkephalin sequences could be identified in brain extracts, and progress was being made in identifying the very different basic opioid peptide fraction isolated from pituitary by Goldstein and others. The breakthrough for the enkephalin precursor came when it was found that adrenal medullary tissue offered a good source for additional studies on ME precursor peptides (21). Eventually a novel precursor, pro-enkephalin, was isolated from adrenal medulla [for a summary of these studies, see (22)]. This precursor peptide was found to contain six copies of the ME sequence and one copy of the LE sequence. Pro-enkephalin is now known to be the primary precursor for ME and LE. The gene sequence for pro-enkephalin was soon discovered by the Numa (Kyoto), Udenfriend (New Jersey) and Herbert (Oregon) groups, all working independently (23–25). A partial sequence of the highly basic opioid peptide from pituitary isolated by the Goldstein group was published in 1979 (26), with the full 17-amino acid peptide sequence of the peptide, named dynorphin, finally sequenced by Goldstein in 1981 (27). Two closely related peptides named α- and β-neoendorphin were isolated from porcine hypothalamus in 1981 (28, 29). When the sequence of the gene coding for pro-dynorphin was finally determined, again by the Numa group, in 1982 (30) it was apparent that, like the proenkephalin gene, the prodynorphin gene also encoded several closely related opioid peptide sequences, dynorphin A, α-neoendorphin, and dynorphin B, that are liberated from the precursor peptide by the actions of selective endopeptidases. For a fuller discussion of the processing and properties of the active opioid peptides derived from the POMC, proenkephalin and prodynorphin genes, see Fricker et al. (31).

These developments revealed a complexity in the endogenous opioid system that was certainly not expected when the search for the natural regulators of the postulated opioid receptor was initiated in the early 1970s. Processing of proenkephalin and prodynorphin was shown to generate several biologically active peptides from each precursor, leading to studies over the coming decades on the peptide processing enzymes and the predominant active forms of the peptides in different tissues and brain regions. INRC meetings at this time contained many reports of the activities of various opioid gene products in bioassays or when injected into discrete brain regions, while others evaluated the degradation of these peptides, and it was demonstrated that β-endorphin in particular could be identified circulating in plasma in animals and in man. In contrast, ME and LE are so rapidly metabolized by tissue enzymes that these peptides do not circulate in blood at physiologically relevant concentrations. A recent review (31) has summarized the development of understanding of peptide processing machinery and the implications of this complexity in relation to the functions of endogenous opioids. The decade between 1975 and 1985 also saw the identification of another group of opioid peptides, the casomorphins, derived from digestion of β-casein in milk (32). β-casomorphins derived from breast milk appear to play a role in regulating intestinal motility and secretions in neonates (33, 34). A dipeptide (Tyr-Arg) named kyotorphin was identified in brain by (35). Kyotorphin does not appear to interact directly with opioid receptors but may affect the release or stability of released enkephalins (36). The actual peptide level and physiological relevance of the multitude of different proenkephalin and prodynorphin-derived peptides is still poorly understood and understudied. A recent paper by Lakshmi Devi and others determined affinity, activity, and potential ligand bias at each opioid receptor for the vast majority of these endogenous peptides and found there to be considerable overlap, with endogenous ligands derived from each prohormone able to activate each opioid receptor at physiological concentrations (37). Nevertheless, at this time the physiological actions of most of the opioid peptides produced are not known.

### Opioid Receptor Heterogeneity and Signal Transduction Pathways

During the period from 1973 to 1976, while many groups were giving most attention to the search for “the endogenous opioid,” others continued to explore the pharmacologic properties of the rapidly expanding arsenal of opioid drugs. Martin and others, at the Addiction Research Center in Kentucky, in particular, developed a novel experimental model using dogs that had been chronically decerebrated to permit the analysis of the effects of drugs on spinal and brain stem reflexes without the modifying functions of descending inhibitory systems. In this model, Martin found that the actions of several benzomorphan analogs were qualitatively different from those of morphine. Expanding on his earlier “receptor dualism” model, he now suggested that these results were best explained by the presence of three distinct opioid receptor types. One group of symptoms in his dog preparations, including miosis, bradycardia, hypothermia, and depression of nociceptive responses, was produced by morphine and closely related drugs. Another cluster of actions, including miosis, depression of the flexor reflex and sedation but with little effect on pulse rate or the skin twitch reflex, was associated specifically with the benzomorphan ketocyclazocine, while a third set of actions, including mydriasis, tachypnea, tachycardia, and mania, was uniquely induced by a related drug, SKF 10,047 (N-allylnormetazocine*)*. Martin proposed that three receptor types were responsible for these different sets of actions, and named the receptors by the Greek symbols for the first initial of the most characteristic drug activating each site; μ (mu) for the morphine, κ (kappa) for ketocyclazocine and σ (sigma) for SKF10,047. This work was published in 1976 in a seminal paper proposing the existence of three distinct forms of receptors for opioid drugs (38).

Martin’s paper in the Journal of Pharmacology & Experimental Therapeutics was quickly followed by a new study from the Kosterlitz group. By 1977 they had realized that the target sites preferentially activated by ME and LE differed significantly from the target sites for morphine and close analogs in isolated tissue bioassay preparations, with respect to their relative apparent potencies for opioid ligands. The target sites preferred by morphine, well represented in the guinea pig ileum assay, appeared to be similar to Martin’s μ receptor, while ME and LE showed significantly higher apparent potency relative to morphine for the receptor predominant in the mouse vas deferens preparation that had been instrumental in their initial discovery of the enkephalins. They labeled the receptor in the mouse vas deferens preparation the δ (delta) receptor (after the potent metabolically stable enkephalin analog, [D-Ala^2^, D-Leu^5^]enkephalin (subsequently known informally as DADLE), that was a potent activator of this site (39). In the same study they also showed that radiolabeled morphine or DADLE binding to isolated brain membranes could be distinguished by the relative affinities of morphine and enkephalin analogs in displacement studies.

This report, together with the Martin’s 1976 paper, triggered a profound discussion on the apparent existence of multiple receptors for opioid analgesic drugs and their relative potential as therapeutic targets. The multiplicity of opioid receptors was not immediately accepted by all in the field. The experimental approach used by Martin and others was subject to alternative explanations, was unique to his laboratory, and did not use techniques that had traditionally been employed by pharmacologists to define differences among receptor types, raising concerns for some investigators. Kosterlitz had used the apparent affinities of an antagonist (naloxone) to distinguish δ from μ receptors (a classic pharmacologic technique), but his model relied mainly on studies in isolated tissues; some investigators argued that the δ receptor had not yet been shown to play a role in CNS function.

This controversy continued to fill the programs of INRC meetings from 1976 onwards for a number of years and was probably a major incentive for many investigators to attend these meetings. It was noteworthy that a significant fraction of INRC meeting attendees over the next decade or more came from the private sector, as the drug industry tried to keep up with developments in the field and to determine if the proposed novel receptors might be effective therapeutic targets. The 1978 meeting, held in July at Noordwijkerhout, a somewhat isolated resort site on the cold and windy North Sea coast of the Netherlands, attracted more than 250 registered attendees; the proceedings of this meeting were also published as a monograph (40).

Over the next few years studies confirming the heterogeneity of the receptors activated by opioids drugs quickly followed, confirming the early insights of Martin and Kosterlitz. It was quickly discovered that the receptor activated by morphine in NG108-15 cells, demonstrated by Werner Klee and Marshall Nirenberg in 1974, had much higher affinity for DADLE relative to morphine than was observed at the μ receptors, suggesting that this receptor closely resembled the δ receptor proposed by Kosterlitz (41). This would turn out to have great significance in another decade leading to the cloning of the first opioid receptor. Others showed that ketocyclazocine produced effects *in vivo* that were clearly different from those of morphine, while radiolabeled ketocyclazocines bound to a site in brain membranes that differed from the preferential binding sites for morphine. In 1981, Chavkin and Goldstein used the apparent relative affinities for naloxone in antagonizing different opioid peptides and opioid drugs in the guinea pig ileum preparation, and the selective protection of subsets of receptors from irreversible blockade by the μ-selective irreversible antagonist β-FNA, to show that dynorphin was an endogenous ligand for κ-receptors in this tissue, while morphine acted through μ receptors also expressed in the tissue (42, 43). As a result, by the early 1980s there was general agreement that μ, δ and κ receptors existed, each having different functional roles in the central nervous system and in peripheral tissues, and the commonly used name for the receptor family changed from “opiate” to “opioid,” reflecting the general understanding that this receptor family serves as physiologic targets for the three families of opioid peptides. These general conclusions were eventually justified by the discovery of genes coding independently for the μ, δ and κ receptors in the early 1990s. The existence of the σ receptors initially proposed by Martin et al (38) remained uncertain. While Martin and others had reported that the actions of SKF-10,047 were antagonized by nalorphine, others were not able to replicate antagonism of the actions of SKF-10,047 by opioid antagonist drugs. Ultimately it became apparent that the racemic SKF-10,047 studied by Martin’s group contained two stereoisomers with different pharmacologic and behavioral properties; (−)SKF-10,047 had high affinity for μ and κ receptors and could be antagonized by naloxone, while (+)SKF-10,047 had high affinity at a non-opioid σ-receptor, as well as the PCP site on the NMDA receptor, and was not sensitive to antagonism by naloxone or other opioid antagonists (44). It is now clear that σ-receptors are functional in the CNS but they are not part of the opioid receptor family and are not activated by endogenous opioid peptides.

INRC meetings through this decade also saw advances in understanding of the signal transduction pathways activated *via* opioid receptors. Other groups confirmed the original work of Collier, Nirenberg and Hamprecht from the mid-1970s showing that opioids could inhibit AC activity in cell lines and in brain, but more complete understanding of this system required a greater understanding of the mechanisms underlying receptor regulation of AC. A critical development was the demonstration by Martin Rodbell (45) that a novel nucleotide-binding protein (now called Gi) was required for inhibition of AC by activation of several neurotransmitter receptors including opioid receptors. With the identification of the transduction role of G proteins, a flurry of studies followed, further characterizing opioid receptor regulation of AC as a primary signaling mechanism for all three types of opioid receptor, and identification of direct regulation of ion channels by G protein subunits followed. As the number of isoforms of G protein alpha, beta and gamma subunits increased there was an increasing appreciation of the complexity of the signaling pathways regulated by opioids. Electrophysiologists had previously demonstrated that opioids could increase potassium conductance in neurons (46) and later studies demonstrated opioid-mediated inhibition of some calcium conductances by mechanisms requiring G protein signaling, thus providing a new understanding of the role of G protein activation as a critical regulator linking opioid receptors to both metabolic and ionic regulation of cell activities. Klee and Nirenberg (17) had also reported that chronic exposure to opioid drugs resulted in an increase in AC activity in NG108-15 cells, proposing that this adaptive modulation of a down-stream signaling system provided an experimental confirmation of a theoretical model for drug tolerance and dependence that had been proposed much earlier by Goldstein (47) and independently by Shuster (48).

### Opioid Peptides and the Search for Better Analgesic Drugs

The primary focus of INRC meetings during the first two decades was in understanding the nature of the endogenous opioid system, its distribution across the brain, spinal cord and peripheral nervous systems and how this system responded to chronic activation by exogenous opioid drugs, but the goal of achieving improved analgesic therapies was not forgotten. Most meetings included contributions from medicinal chemists reporting on novel synthetic structures with opioid-like properties. Leading contributors to the field, including Paul Janssen, the inventor of fentanyl and John Lewis, a member of the group that first reported the extraordinary potency of etorphine and other oripavine derivatives, attended a number of the early meetings. Also of critical importance was the discovery by scientists at Upjohn of a series of highly selective κ agonists (49) that have been widely used for the characterization of most κ activities, as well as binding studies. Academic opioid chemists, including Kenner Rice and Phil Portoghese greatly enriched opioid pharmacology. In particular, Portoghese designed the first selective κ antagonist norBNI (50) as well as the irreversible μ antagonist β-FNA (51), both of which have been indispensable in understanding and characterizing the opioid system.

With the determination of the structures of endogenous opioid peptides, the pharmaceutical industry committed considerable resources to developing synthetic peptides and other small molecules that would retain the high efficacy at opioid receptors of the endogenous peptides, but with increased metabolic stability and access to the central nervous system after systemic administration. The focus in these sessions continued to be on the actions of endogenous opioids, chemically protected opioid peptides and novel small molecule drugs in pre-clinical models for evaluation of pain relief. The hope was that modified peptide structures might be effective analgesics with reduced addiction liability. Many novel peptides were identified, and some, in particular the highly selective μ ligand DAMGO ([D-Ala^2^, *N*-MePhe^4^, Gly-ol]-enkephalin) (52) and the δ ligand DPDPE ([D-Pen^2^, D-Pen^5^]enkephalin) (53), both metabolically stable enkephalin analogs, proved to be very useful in discriminating different opioid receptor types, but none of the novel agents developed at this time was an effective analgesic that could replace morphine or any of the other established opioid analgesic drugs.

A number of INRC meetings during this period were held as back-to-back meetings with CPDD, sometimes with overlapping sessions on topics of common interest. Generally, clinical studies of novel analgesics along with evaluations of addictive liability were more likely to be presented at CPDD than at INRC conferences. Eddie Leong Way, who chaired the pharmacology department at the University of California San Francisco and made many early contributions to opioid pharmacology, took over as Secretary/President in 1984, overseeing meetings in Cambridge UK (1984), Seacrest in Massachusetts (1985), San Francisco (1986), and Adelaide, Australia (1987). In 1988 Albert Herz (Max- Planck, Munich), one of the INRC Founders, who became the first non-USA-based Secretary (after the first two INRC meetings organized by Hans Kosterlitz), organizing meetings in Albi France; St. Adele, Canada, and Noordwijkerhout, Netherlands before handing the role to Huda Akil (University of Michigan) who became the first woman to serve as Secretary (later called President) of the Conference.

### Opioid Receptor Cloning

While the genes coding for each of the three major families of endogenous opioids had been identified and sequenced fairly quickly, identifying the genes coding for what were now generally agreed to be three different opioid receptor isoforms proved more difficult. Their level of expression in neural and other tissues appeared to be very low and their isolation and therefore sequencing required for cloning was challenging. By the end of the second decade of INRC’s history, in 1990, a number of groups had active programs seeking the critical genes, but success was elusive.

The first mammalian GPCRs cloned were bovine rhodopsin (54) followed by Lefkowitz and others in 1986, after a Herculean effort to purify and sequence a portion of the β_2_-adrenergic receptor sufficient to screen a hamster genomic library (55). Cloning of other GPCRs followed and by 1992, opioid receptors had still not been cloned, despite considerable effort from many labs. There were some false starts, including the cloning of a receptor that didn’t turn out to be opioid (56), Finally, this changed near the end of 1992 when two papers came out nearly simultaneously by Chris Evans (57) and Brigitte Kieffer (58) describing the expression cloning and sequence of the δ opioid receptor from NG108-15 cells. When transfected into COS cells, both groups described the expected 7-transmembrane protein with high affinity for the known δ opioid receptor ligands that inhibited cAMP accumulation, and the actions of these ligands were blocked by naloxone. Because of sequence homology among the opioid receptors, the cloning of other opioid receptor genes followed rapidly. The κ receptor was cloned by Graeme Bell (59), while he was looking for a novel somatostatin receptor, and this was quickly followed by cloning of the μ receptor independently by Lei Yu and George Uhl (60, 61). These developments led to considerable excitement at the 1993 meeting in Skõvde, Sweden when the data on each of the opioid receptor types was discussed, opening a new era of opioid research with the receptors being studied now in transfected cells without the complication of multiple receptors being present in the tissue preparation.

There were several surprises upon cloning of the opioid receptors. For one thing, there were only three, δ, κ, and μ. Previous binding and *in vivo* studies had putatively identified subtypes for each isoform, μ1, μ2 (62); μ3 (63); δ1, δ2 (64); at least four κ receptors (65); and even more subtypes were proposed by the most enthusiastic investigators. However, no matter how hard people looked for additional genes for receptor subtypes or splice variants that could adequately explain the previously described receptor heterogeneity, the genes, or appropriate splice variants, just didn’t exist. This is in contrast to the serotonin receptor family, for example, that had two or three described isoforms prior to cloning, and ultimately was found to consist of a full 15 different 5-HT receptor genes. While the apparent presence of multiple opioid receptor subtypes did not have a genetic basis, the other main surprise from the receptor cloning studies was the identification, by several researchers, of another closely related receptor with a pharmacology that was distinctly non-opioid. This receptor named by various groups ORL1, LC132, XOR1, kappa 3, ROR-C, C3 had sequence homology to μ, δ, and κ receptors that was nearly as high as they had with each other, but when transfected into mammalian cells, the novel receptor was found not to be activated by most opioid ligands with high affinity for the other opioid receptors (66–71). Meunier and others, who named it **O**pioid **R**eceptor **L**ike 1 (ORL1) presented data at INRC in 1994 at the Seacrest Hotel in North Falmouth, MA, demonstrating that a few opioid drugs such as etorphine also inhibited cAMP accumulation in cells expressing only ORL1 receptors, with modest (30 nM) potency. However, this activity was not reversed by naloxone at reasonable concentrations, excluding this receptor from being described as opioid based on pharmacologic criteria. Nevertheless, sequence homology, gene structure and a common signal transduction mechanism clearly puts this receptor in the opioid receptor family (72).

The identification of the fourth opioid-family receptor initiated the search for the endogenous ligand, since the previously identified opioid peptides had very low affinity for the fourth receptor. A novel endogenous ligand for ORL1 was identified the following year by Meunier and others (73) and simultaneously by Civelli and others (74). These results were presented by Jean Claude Meunier at the 1995 INRC meeting in St. Andrews, Scotland. This 17-amino acid peptide, called nociceptin (by Meunier et al.) and orphaninFQ (by Reinscheid et al.), was remarkable in that it had the N-terminal opioid peptide sequence X-Gly-Gly-Phe, where the X was Phe rather than Tyr, which is found in the enkephalins, β-endorphin and dynorphin. It had been well established that the OH on Tyr was required for binding to the original three opioid receptors, explaining its lack of affinity for these receptors and offering a strong clue as to why opioid peptides did not bind ORL1. This neuropeptide is now officially named N/OFQ and the receptor is named the NOP receptor (Nociceptin/Orphanin F/Q Opioid Peptide receptor).

Nociceptin/Orphanin FQ was not the only new opioid ligand discussed at INRC meetings during this period. In 1997 Jim Zadina and others identified two novel peptides they had found in extracts of bovine frontal cortex (75). These peptides, called endomorphin-1 (Tyr-Pro-Trp-Phe-NH_2_) and endomorphin-2 (Tyr-Pro-Phe-Phe-NH_2_) had high affinity and very high selectivity for the μ opioid receptor. This was important since the known opioid ligands (enkephalin, dynorphin, β-endorphin) each had significant affinity and efficacy at more than one opioid receptor type, indicating that it was unlikely that release of one endogenous opioid would activate only one receptor type. The high μ-selectivity of endomorphins suggested that they may be selective endogenous μ-receptor agonists. Selective ligands would greatly simplify our understanding of receptor activation if we could identify the relative localization of the receptor and ligand. Upon identification of the endomorphins the search for the gene(s) coding for these peptides began in many labs. However, to this day, no gene has been identified, leaving the distinct possibility that these ligands don’t exist *in situ* and therefore they remain putative μ receptor endogenous ligands.

Upon receptor cloning, the next obvious innovation was the development of knockout animals to verify the necessity for opioid receptors for opioid-mediated actions. The first mouse with an opioid receptor deleted by homologous recombination, naturally, was the μ receptor, the receptor type most important for both analgesia and drug abuse, which was first discussed at the 1997 INRC meeting, in Hong Kong. These mice, developed in the labs of Brigitte Kieffer and George Uhl were healthy and developed and bred normally (76, 77). As expected, morphine-induced analgesia, as well as morphine reward were abolished, proving conclusively that the μ-opioid-receptor gene product is the molecular target of morphine *in vivo*. Additional knockout mice for all four receptors in the opioid receptor family were produced in the labs of Brigitte Kieffer and John Pintar and were made available to be used by the entire opioid community. One other important result of the knockout studies was the verification that there were only the three opioid receptors (μ, δ, κ) and deletion of all three genes (triple knockout) eliminated all opioid binding activity (78).

Although it had been proven that the genes that encoded μ, δ, and κ receptors were sufficient to explain basically all opioid activities, including those of subtype selective ligands, the *in vitro* and *in vivo* pharmacology that initially resulted in the description of these subtypes now required an explanation. One possible factor leading to the expression of functionally different receptors from a single gene was the possible tissue or cell-type specific expression of splice variants formed during transcription of the gene. Gav Pasternak and others have been at the forefront in the evaluation of this possibility. Initially using a technique that they called “antisense mapping,” first discussed at the 1998 meeting in Garmisch-Partenkirchen, Germany, Pasternak and others used antisense nucleotides to knock down specific receptor gene exons and found that this was sufficient to differentiate the actions of morphine versus heroin (79, 80), perhaps explaining the binding and functional differences between μ1 and μ2 receptors that Pasternak had so long discussed. Over the ensuing years Pasternak and his colleague Ying-Xian Pan delved in great detail into the μ receptor describing a multitude of splice variants, presented at multiple INRC meetings. At this point, they have described at least 12 potential splice variants, including a 6 transmembrane version that binds opioids with its own specific pharmacology (81, 82). The physiological significance of this potentially large number of different μ receptor isoforms is still not known and still under investigation.

### Visualizing Sites of Receptor Expression

One of the major benefits of cloning the receptors was the ability to visualize them. Although *in vitro* autoradiography had been utilized for more than 10 years, and was quite successful, the resolution was only sufficient to obtain a very general regional localization of a receptor. Cloning led to two advancements, the gene and therefore the RNA sequence was known, which could be used for *in situ* hybridization, and the receptor, or fragments of the receptor could be purified or synthesized to make antibodies for immunohistochemistry. These techniques, which had been used for identification and localization of opioid peptides for the past 10 years, revolutionized receptor localization and allowed for the cellular, rather than regional, identification of opioid receptor expression. These techniques were used widely, with the leading proponents in the opioid field being Stan Watson and Huda Akil. Watson and Akil, characterized locations of each of the opioid receptors, and NOP, and compared the localization of the receptor with its endogenous ligand (83–86). These studies were presented at multiple INRC meetings. On the other hand, complications arose with the immunohistochemical studies on opioid receptors that reflected the challenges of working with receptor antibodies. It turned out that many of the opioid receptor antibodies, including many commercial products, were apparently non-selective and when staining on knockout mice was used as a control, a similar pattern of localization could be recognized in the wild type and knockout animals. Validation that there is no immunohistochemical staining in knockout animals is now required for appropriate rigor when utilizing opioid receptor antibodies.

### Developments in Understanding of Signal Transduction Pathways

Throughout the 1990s and early 2000s, a continuously important topic at INRC meetings pertained to the signal transduction mechanisms of opioid receptors. This is a particularly important topic for opioid receptors because of the presumed relevance to all aspects of opioid actions, including analgesia, reward, addiction, tolerance development, etc. By this time, it had been many years since Rodbell and Gilman had described and purified G proteins, but the additional components of signal transduction pathways and the importance of receptor phosphorylation were being described. These were not exclusive to opioid receptors, of course, but were very important to the development of the field and formed a major topic of discussion at INRC meetings. The steps after receptor binding, including receptor phosphorylation by G Protein Receptor Kinase (GRK), binding of β-arrestin, MAP kinase activation, internalization and recycling take place in the seconds to minutes after agonist binding. An excellent review of μ receptor regulation by a group of INRC members describes many aspects of μ receptor changes at the biochemical and cellular level and reflect what was discussed at many INRC meeting (87). Much of the work, from the group of Graeme Henderson demonstrated, using electrophysiology, that μ receptor activation leads to phosphorylation and rapid desensitization in a variety of neurons (88, 89). This was discussed in his Founders Lecture at INRC 2013 in Cairns, Australia. Additional studies by Mac Christie (90, 91) and John Williams (92, 93) in the locus coeruleus and the vlPAG, also presented at multiple INRC meetings, have characterized the kinases involved in phosphorylation as well as the kinetics of desensitization and recovery. Stefan Schulz and others have further characterized the critical phosphorylation sites on opioid receptors (94, 95). Downstream signaling through MAP kinases has also been described in detail. An additional downstream signaling cascade involving phosphorylation of c-jun kinase (JNK) induced by both antagonists and agonists, originally proposed by Chavkin and Bruchas (96, 97), was discussed at multiple INRC meetings and appears to be involved in inactivation of Gi-coupled GPCRs, potentially leading to tolerance development induced by agonists, and the long-lasting actions of κ antagonists, such as nor-BNI and JDTic. Despite the large number of studies identifying signal transduction parameters, there is still much more to learn of the interactions among different down-stream signaling pathways following activation of opioid receptors.

One major topic in our attempts to understand the action of opioids pertains to the rewarding and addicting nature of the compounds. Why are opioids abused, where do they work, why is it so hard to stop, and why is relapse so prevalent? The dopamine hypothesis of drug reward suggested that reward is induced by an increase in extracellular dopamine in the Nucleus Accumbens mediated by excitation of dopaminergic neurons in the ventral tegmental area (VTA). Alan North demonstrated that activation of opioid receptors on GABAergic interneurons in the VTA disinhibited the dopaminergic projection neurons leading to increased dopamine release (98). The involvement of opioids in this pathway has been studied using electrophysiological techniques by a number of INRC participants, in addition to North, including John Williams, Howard Fields, and Elyssa Margolis (99–101). Groups headed by Toni Shippenberg and Mary Jeanne Kreek measured dopamine directly using microdialysis to examine the relationship between dopamine and behavioral responses (102, 103). But the inverse to dopamine reward has an equal influence on drug-taking. It has been known since the 1930s that the strongly aversive aspects of opioid withdrawal are a significant factor in return to illicit drug use during the period of withdrawal. At a Plenary Lecture in at INRC 2002 in Monterey CA, George Koob addressed the dark side of drug addictions in general, describing specifically a plasticity of the kappa opioid system that is induced by chronic opioid use, and in chronic alcoholism. Studies by his group, as well as Mary Jeanne Kreek and others, clearly demonstrated that psychostimulant abuse increase dynorphin levels (104, 105), while Charles Chavkin and others demonstrated that stress-induced relapse was due to κ receptor activation (106, 107). Dynorphin and κ receptor activation in general, is dysphoric, as first demonstrated by Albert Herz (108, 109). Because of the upregulation of the κ system, progressively increased drug use is required to maintain a normal hedonic state, while discontinuation leads to κ-mediated anhedonia and dysphoria, a condition that Koob named “hyperkatifeia,” which he further described in a Plenary Lecture at INRC 2019 in New York. Ultimately, the interplay between μ and κ systems contribute to many aspects of drug abuse including acquisition, withdrawal and relapse.

### Receptor Dimerization

A persistently controversial topic at INRC and in the GPCR field in general pertains to receptor homo- and heterodimerization, and higher levels of oligomerization. There had been suggestions by Eric Simon and others that opioid receptors could be physically associated based upon crosslinking studies with the non-selective ligand [^125^I] β-endorphin (110). The first direct evidence of opioid receptor dimerization was obtained by Lakshmi Devi and others, who demonstrated co-immunoprecipitation of differentially (Flag and c-Myc) epitope-tagged δ opioid receptors (111). This work was presented at the 1996 meeting in Long Beach, CA. The ability to dimerize was not limited to homodimers, or even GPCR dimers within the opioid receptor family. Susan George, who worked mostly on dopamine receptors, produced μ/δ dimers in COS cells and demonstrated differential pharmacology of opioid ligands, discussed at the 2003 INRC meeting in Perpignan, France (112). Devi produced considerable additional evidence for opioid homo- and heterodimers, and multimers, as well as δ/CB1 dimers. She later identified both ligands (113) and antibodies (114) that were uniquely selective for opioid receptor dimers. These experiments were discussed at several INRC meetings in the early 2000s. The promise of opioid receptor dimers was that dimer-selective compounds could be identified that might improve selectivity of opioid ligands, potentially reducing side effects and perhaps tolerance development. This goal has not yet been realized with dimer-selective agents, and a major focus of groups seeking to develop analgesic drugs with improved side effect profiles shifted during the early 2000s to a different important topic, ligand bias.

### Biased Agonism and Relative Efficacy

The field and implications of ligand bias started with Laura Bohn, when she was a postdoc for Marc Caron, with her demonstration that a strain of β-arrestin 2 knockout mice treated with morphine demonstrated potentiation and prolongation of analgesic responses to morphine, with little or no development of tolerance, work initially presented in 2000 at INRC in Seattle (115, 116). At this meeting, future Nobel Prize winner Bob Lefkowitz gave a Plenary Lecture on β-arrestin and discussed the implications of designing compounds that did or did not activate this enzyme. In Bohn’s original studies, G-protein activation appeared critical for analgesia while β-arrestin signaling appeared to be specifically associated with respiratory depression (Bohn et al, 1999). Subsequent studies demonstrated that the β-arrestin 2 knockout mice displayed increased extracellular dopamine and increased conditioned place preference in response to morphine treatment (117). These early results, presented at multiple INRC meetings led to the hypothesis that some opioid agonists could selectively activate individual signal transduction pathways, activating either G-protein-dependent or β-arrestin-dependent signaling pathways, in other words, biased agonism, which could give an improved profile of analgesic activity relative to side effects. This is a very attractive hypothesis and suggests that one might be able to design compounds that have potent analgesic activity without the μ-mediated side effects of respiratory depression, constipation, and reward/abuse liability. This hypothesis led to countless studies examining bias of known ligands, the involvement of medicinal chemists to design and synthesize “biased” agonists, and even the founding of a company (Trevena), whose business model was the design of biased agonists for the development of opioid analgesics, with reduced side effects. Many of these studies were presented at INRC over the ensuing 15 years. This included a talk by Ashish Manglik, from Nobel Prize winner Brian Kobilka’s lab, at INRC 2016 in Bath, England, and his Young Investigator Award lecture in 2017 in Chicago, who used computational methods to identify a biased mu agonist (PZM21) that had analgesic activity with reduced respiratory depression and reward (118). A subsequent lecture by Jonathan Violin of Trevena, demonstrated, at INRC 2017 in Chicago, that biased agonist TRV130 (oliceridine) had potent analgesic activity and reduced respiratory depression, and therefore an apparently wider therapeutic window than morphine through Phase 3 clinical trials. Oliceridine ultimately failed to show significant differentiation of analgesic versus respiratory depressant activity in clinical trials but has been approved for use in humans, with an i.v. formulation, and represents perhaps the newest novel opioid structure approved for use in humans.

Ultimately the extent of bias of PZM21 and other agonists was questioned by several labs and a talk by Alexander Gillis, from Mac Christie’s lab at INRC in 2018 in San Diego, suggested it was its relatively weak partial agonist activity, rather than a lack of recruitment of β-arrestin, that mediated the reduced respiratory depression of “biased” agonists (119). Studies from the labs of Stefan Schulz, Macdonald Christie, Graeme Henderson and others have demonstrated that many of the most G-protein-selective ligands have low efficacy relative to morphine (and very low efficacy relative to fentanyl) at μ receptors and furthermore, that in mice with modified μ-receptors unable to recruit β-arrestin, opioids still induced respiratory depression (119, 120). Consistent with these findings, studies in three laboratories demonstrated that morphine-induced respiratory depression was not eliminated by genetic deletion of β-arrestin-2 (121). These results suggest that relative efficacy is more likely to be the major factor in determining the ratio of analgesic to respiratory depressant activity among opioid drugs. Presently, this issue is not resolved, and ligand bias may still play a role in the development of drugs with fewer side effects.

### Crystal Structures

In 2007, the crystal structure of the β_2_-adrenergic receptor was published by Brian Kobilka and others. This remarkable accomplishment, which required the presence of a receptor-bound inverse agonist and a monoclonal antibody to stabilize the third intracellular loop, was followed, over the next several years, by similar procedures to crystalize a large number of other GPCRs, by his group and by Ray Stevens and others. Among the early GPCRs to be crystalized were all the receptors of the opioid receptor family, which were published independently 2012 (122–125). There was nothing particularly noteworthy about these receptors, compared with other GPCRs, but the three-dimensional structures did confirm changes in the binding pockets that lead to similarities among the receptors and particularly differences in ligand binding leading to the divergence of the NOP receptor from the classical opioid receptors (μ, δ, and κ) (124). Naturally, the crystal structures provided a level of receptor scrutiny that was previously unavailable. These crystal structures, and additional agonist-bound crystal structures (126, 127) allowed for a better understanding of an agonist-induced conformational change, an intramolecular interpretation of the sodium binding site and the sodium effect, and structures for in silico docking of old and novel ligands to the receptors. At the 2017 INRC, Marta Filizola and others discussed molecular dynamics studies based upon the opioid receptor crystal structures to identify potentially druggable stable and metastable receptor states, and the μ receptor crystal structure was used for the identification of novel potentially biased agonists, such as PZM21, discussed at INRC in 2016 and 2017. These and other medicinal chemistry studies described at INRC in 2018 and 2019, demonstrate the natural progression of pharmacological concepts, describing the interaction of opioids with their receptors to the angstrom level resolution of the receptors used to define the interaction of the receptor with its surroundings and endogenously or exogenously delivered opioids.

### New Ligands for Opioid Receptors, and New Research Tools

As noted earlier, medicinal chemistry has always been an important component of INRC. In the early days, chemists were making novel opioid ligands as potential analgesic drugs and for use in defining the properties of μ, δ, and κ receptors. Irreversible ligands were designed, initially for the purification of the receptors, though they were never really useful in that endeavor, but they were valuable in establishing the existence of an excess of opioid receptors (“spare” receptors) and determining intrinsic efficacy of opioid ligands, in neural pathways mediating analgesia (128). Over the past 20 years, as the concepts have changed, so have, the goals of the medicinal chemists. With the understanding of the involvement of kappa receptors in withdrawal-induced anhedonia and dysphoria, κ receptor antagonists became a target as potential medications in the treatment of drug addiction. Novel agents included a series of high affinity and potent κ antagonists, designed by Ivy Carroll, including a compound called JDTic, first presented at INRC 2011 in Hollywood, FL. JDTic acted much like norBNI, with κ antagonist activity lasting for weeks in animal models (129, 130). In animal models, JDTic showed potent and long-lasting blockade of stress-induced cocaine relapse (131), and was developed by NIDA for treatment of cocaine use disorder. Unfortunately, the clinical trial lasted only a few weeks, and as described at INRC 2015 in Phoenix, AZ, a very mild cardiac arrythmia in a single patient led to discontinuation of further clinical studies with this compound. Subsequent to the identification of JDTic, several selective short acting κ antagonists have been described, both peptides from Jane Aldrich (132) and non-peptides from Eli Lilly and Blackthorn Therapeutics (133, 134) have been identified. These compounds have shown some efficacy for patients with treatment-resistant depression (135). More recently, there has been a flurry of reports of novel compounds based upon previous discoveries, including the discovery of the natural product, salvinorin A, as a high affinity and highly selective κ agonist, reported at INRC 2008 in Charleston, SC. This compound, from a plant (Salvia divinorum) found in southern Mexico, is particularly unusual among opioid receptor ligands in that it contains no basic nitrogen, previously considered to be essential for binding to opioid receptors. Tom Prisinzano and others worked with this compound to design novel structures that are reported to demonstrate exquisite bias towards G protein rather than β-arrestin signaling (136, 137). These might be useful as analgesics without the dysphoria associated with non-biased κ agonists. Other chemists including Chris McCurdy and Sush Majumdar, have worked with other natural products including the primary active agent of kratom, mitragynine, to develop novel G-protein-biased agonists with significant analgesic activity and reduced respiratory depression and physical dependence (138–140). It’s possible that ligand bias is more relevant to κ-mediated analgesia than μ-selective ligands. Steve Husbands and Nurulain Zaveri have synthesized NOP/μ partial agonists that seem to have potent analgesic activity in non-human primates without any apparent normal μ side effects (141, 142). Finally, Phil Portoghese, at 90, is still producing novel bivalent ligands targeting μ opioid receptor activation together with δ (143) or mGluR5 antagonist activity (144), or together with CB1 agonist activity (145), all with beneficial analgesic properties. It remains to be determined if any of these novel agents will eventually make a significant contribution to the treatment of pain and drug abuse disorders.

Finally, INRC is still a venue for reporting developments at the cutting edge of neuroscience, with reports of a variety of new tools that are available for all. From fluorescently tagged δ, μ, κ, and NOP receptors produced by Brigitte Kieffer, Lee-Yuan Liu-Chen and Michael Bruchas (146–149), to floxed and cre opioid receptors and peptides (150–154), genetic models are being developed to explore opioid circuitry with respect to pain and drug abuse. New detection methods with exquisite spatial resolution such as mini-microscopes that can be mounted on freely moving animals, (Michael Bruchas, Greg Scherrer), microdialysis paired with liquid chromatography/mass spectrometry as well as micro-immunoelectrodes to detect opioid peptides (Ream Al-Hassani) and *in vivo* fast scan voltammetry (Leslie Sombers) were all discussed at our last in person meeting, the 50th INRC in New York in 2019. With a dedicated group of young scientists, we expect innovation in this vast and fascinating field to continue.

### What has Been the Impact of the INRC?

Quantitative indicators of the contributions of INRC to understanding in this interesting and clinically relevant area of pharmacology are hard to identify. The opioid system has turned out to be incredibly more complicated than originally anticipated by the Founders just over 50 years ago. Many of the new developments were reported very quickly at INRC meetings, justifying the goal of the Founders in creating a venue where all interested researchers from many different biomedical disciplines had an opportunity to share new developments rapidly with colleagues, to offer and receive constructive criticism from multiple perspectives, and to quickly establish collaborative studies evaluating, extending, and exploiting the new discoveries. The new discoveries would probably eventually have been made in the absence of INRC, but the opportunity that the early INRC meetings provided for rapid communication among groups located across the world in the era before widespread internet access has certainly accelerated the acceptance or rejection of novel ideas and new experimental approaches.

From its inception, INRC has emphasized the importance of contributions from a wide range of disciplines including medicinal chemistry, molecular pharmacology, biochemistry, neuroanatomy, and behavioral pharmacology, providing a valuable forum for melding the varied contributions to understanding opioid systems and opioid drug pharmacology that they have generated. The content of the meetings has followed the major advances in techniques that have occurred in each of these fields. INRC meetings have also created a self-correcting environment in which novel hypotheses from presenters are encouraged but also subjected to vigorous critical evaluation and sometimes prolonged discussion. Where disputes have arisen, discussion of experimental approaches to resolution of the sources of disagreement have followed. This has been true from the early years of the Conference, when at each meeting Hans Kosterlitz adamantly insisted that any action of a drug that was defined as opioid must be shown to be antagonized by naloxone at relevant concentrations. Numerous issues have been discussed and debated repeatedly in an attempt to reach consensus on the relevance of findings presented at the annual meeting. These important issues include: the role of sodium in modulating opioid receptor function, the evaluation of a physiological role for [Leu^5^] β-endorphin (which is not a naturally occurring peptide), early debates over whether differences in apparent receptor properties represented different conformational states of a common receptor or were evidence of the existence of multiple receptor subtypes, the exclusion of sigma receptors (originally classified as “opioid” by Martin) from the opioid receptor family, the ultimate non-acceptance of the existence of proposed lambda and epsilon receptors, the functional roles (if any) for casomorphins, the existence and biological significance of proposed splice variants for μ- and other opioid receptor genes, the uncertain status of endomorphins as endogenous peptides, and the definition and relevance of ligand bias. These topics, and many others, have all received extensive and sometimes heated discussion at INRC meetings. While these debates have often not resulted in complete agreement by all parties, they have helped in the generation of a general consensus on the critical issues and experimental approaches discussed.

The society continues to emphasize the contributions of young scientists and women in its meetings. While the first meeting had 7% women speakers, the past few meetings have been equally represented by women as attendees, speakers, and travel awardees. In this, it has been greatly helped by the contributions of its donors, particularly an ongoing grant from NIDA, enabling support for young scientists at all meetings. INRC’s emphasis on opportunities for presentation of their work has undoubtedly enhanced the career success of numbers of scientists just entering the field. The international focus of the Conference has led to innumerable useful conversations between members from different continents, a significant number of on-going multi-continental research collaborations, and the widespread acceptance of our rapidly advancing understanding of opioid drug actions, and how these can best be exploited for therapeutic benefit. There is still much to learn, but we hope for a further 50 years of opioid research that generates as much interest, enthusiasm, and excitement at future INRC meetings as we have enjoyed over the last 50 years.
